# Fibrosis-4 Index as a Marker of Systemic Fibrotic Burden and Its Association with Left Atrial Thrombus in Nonvalvular Paroxysmal Atrial Fibrillation

**DOI:** 10.3390/jcm15083063

**Published:** 2026-04-17

**Authors:** Habibe Kafes, Nedret Ulvan

**Affiliations:** Department of Cardiology, Ankara City Hospital, University of Health Sciences, 06800 Ankara, Turkey; nedret.ersoy@outlook.com

**Keywords:** fibrosis-4 index, left atrial thrombus, atrial fibrillation, transesophageal echocardiography, biomarkers

## Abstract

**Background/Objectives:** Left atrial thrombus (LAT) is a clinically important finding in nonvalvular atrial fibrillation (AF). The Fibrosis-4 (FIB-4) index reflects systemic fibrotic burden. We investigated the association between FIB-4 and LAT. **Methods:** This retrospective study included 859 patients with nonvalvular paroxysmal AF undergoing transesophageal echocardiography (TEE). ROC analysis and multivariable logistic regression were performed. **Results:** Left atrial thrombus (LAT) was detected in 10.2% of patients. Patients with thrombus exhibited significantly higher admission FIB-4 scores compared to those without (1.5 vs. 1.1, *p* < 0.001). ROC analysis yielded an optimal FIB-4 cut-off of 1.47 (AUC: 0.65, 95% CI: 0.57–0.70, *p* < 0.001), providing 71.6% sensitivity and 72.0% specificity. After adjusting for CHA_2_DS_2_-VASc score, renal function, and left atrial diameter, a FIB-4 > 1.47 remained a strong independent predictor of LAT (OR: 5.200; 95% CI: 3.105–8.708, *p* < 0.001). However, the addition of FIB-4 to the CHA_2_DS_2_-VASc score did not significantly improve discriminatory performance (*p* = 0.314, DeLong’s test). Spearman’s correlation showed a modest relationship between FIB-4 and CHA_2_DS_2_-VASc (r = 0.321). **Conclusions:** Elevated FIB-4 index values are independently associated with LAT in patients with paroxysmal AF. This simple, noninvasive marker may reflect a systemic fibro-inflammatory milieu that promotes an atrial thrombogenic substrate beyond traditional clinical risk scores.

## 1. Introduction

Atrial fibrillation (AF) remains the most common sustained cardiac arrhythmia in clinical practice, with a prevalence that scales significantly with advancing age [1]. Beyond its symptomatic burden, AF is a primary driver of thromboembolic complications, particularly ischemic stroke and systemic embolism, which continue to be leading causes of arrhythmia-related morbidity [2]. The 2024 ESC Guidelines underscore that left atrial thrombus (LAT), predominantly localized within the left atrial appendage (LAA), is the critical pathophysiological substrate for these events [1]. While transesophageal echocardiography (TEE) is the reference standard for LAT detection, its invasive nature and logistical requirements highlight the clinical necessity for noninvasive, complementary tools to refine the identification of patients at heightened thrombotic risk [3].

The development of LAT is a multifactorial process driven by blood stasis, endothelial injury, and systemic inflammation—conceptually rooted in Virchow’s triad [4]. Although the CHA_2_DS_2_-VASc score is the standard for long-term stroke risk estimation, its precision in identifying active LAT at the individual patient level is often limited [5]. Emerging data suggest that systemic fibro-inflammatory markers may offer additional insight into this thrombogenic environment [6,7,8]. The Fibrosis-4 (FIB-4) index, originally designed for hepatic fibrosis assessment, has recently emerged as a noninvasive surrogate for multisystem fibrotic activity. Beyond liver pathology, elevated FIB-4 levels have been linked to myocardial remodeling, adverse cardiovascular events, and an increased risk of stroke in AF populations, suggesting that it may capture biological risk pathways not fully addressed by traditional clinical scores [9,10,11,12,13].

Despite these established associations with long-term outcomes, the direct relationship between the FIB-4 index and the immediate presence of LAT remains systematically uninvestigated, particularly in patients with paroxysmal AF. Given that FIB-4 is hypothesized to represent a systemic fibrotic burden that may parallel atrial remodeling, it could provide a functional window into the early thrombogenic substrate. Therefore, this study aimed to investigate the association between the FIB-4 index and the presence of LAT in a well-characterized cohort of patients with nonvalvular paroxysmal AF undergoing TEE before rhythm-control interventions.

## 2. Materials and Methods

### 2.1. Study Population

This retrospective study included 984 consecutive patients with symptomatic nonvalvular paroxysmal AF who underwent TEE prior to electrical cardioversion or catheter ablation between December 2019 and April 2024 at a tertiary cardiology center. All patients underwent pre-procedure transesophageal echocardiography to assess for LAA thrombus. Rhythm during TEE was consistent with the procedural indication (AF for cardioversion; AF or sinus rhythm for ablation). Baseline laboratory parameters were obtained within 24 h prior to TEE imaging to ensure clinical synchronization. Patients with valvular AF, defined as moderate-to-severe mitral stenosis or the presence of prosthetic heart valves, were excluded. Patients with persistent or permanent AF, a history of cardiac surgery or catheter ablation for atrial tachyarrhythmias, known liver disease (e.g., viral hepatitis, cirrhosis), active malignancy, hematologic disorders, chronic inflammatory disease, severe renal dysfunction, pregnancy, uncontrolled thyroid dysfunction, or insufficient clinical information were also excluded. Due to the retrospective design of the study and reliance on electronic health records, detailed quantitative data regarding long-term atrial fibrillation (AF) burden—such as the total duration of AF or the exact frequency of episodes detected by prolonged monitoring—were not available for the entire cohort. Consequently, the CHA_2_DS_2_-VASc score was utilized as a clinical surrogate for cumulative thromboembolic risk and atrial substrate remodeling, rather than direct quantification of the specific arrhythmic burden. This limitation was accounted for by focusing the analysis on the independent predictive value of the FIB-4 index within the context of established clinical risk markers. Thus, a total of 859 patients were assessed after applying exclusion criteria. Baseline demographic information, medical history, echocardiographic data, laboratory parameters, risk scores, and medications of the patients at the time of TEE were obtained from the hospital electronic database. The study was in compliance with the principles outlined in the Declaration of Helsinki and received approval from the institutional review board (Decision date: 4 February 2026, decision no: 2-26-1909).

### 2.2. Laboratory Parameters

Peripheral venous blood samples were drawn from a large antecubital vein and collected in EDTA tubes for the hematological tests and dry tubes for biochemistry at baseline. To ensure clinical synchronization with the imaging findings, baseline laboratory parameters required for the FIB-4 index calculation—including aspartate aminotransferase, alanine aminotransferase, and platelet count—were obtained from venous blood samples drawn within the 24 h period prior to the TEE examination. This standardized timing was strictly followed to ensure that the systemic fibro-inflammatory profile reflected the patient’s status at the moment of thrombus assessment. Complete blood counts were measured with an automated hematology analyzer XE-1200 (Sysmex, Kobe, Japan). The baseline FIB-4 index was calculated as: age(years) × aspartate aminotransferase (U/L)/(platelets [109/L] × (alanine aminotransferase [U/L])1/2).

### 2.3. Echocardiography

Echocardiographic data were obtained using transthoracic echocardiography and TEE with a dedicated echocardiography system (EPIQ VCx, Philips Medical Systems, Andover, MA, USA) in a standardized imaging laboratory following current American Society of Echocardiography recommendations [14]. Left ventricular ejection fraction (LVEF) was measured by the modified Simpson method, and left ventricular end-diastolic diameter (LVEDD), left atrial diameter (LAD), and systolic pulmonary artery pressure (SPAP) were also recorded.

All TEE studies were performed by experienced cardiologists in multiple midesophageal projections (0–180°) to comprehensively assess the LAA [15]. LAT was defined as a distinct, well-circumscribed echodense mass within the LA or LAA.

Spontaneous echocardiographic contrast (SEC) was graded visually according to density and flow characteristics. Mild SEC represented minimal, transient echogenicity; moderate SEC was characterized by a persistent, dense swirling pattern; and severe SEC demonstrated marked echodensity with sluggish flow resembling the density of the main cavity [16].

Left atrial thrombus was considered the primary endpoint, while moderate-to-severe spontaneous echocardiographic contrast was also evaluated as a related thromboembolic substrate.

### 2.4. Statistical Analysis

All analyses were performed using the SPSS 27.0 Statistical Package Program for Windows (SPSS, Inc., Chicago, IL, USA). Continuous variables were expressed as mean ± standard deviation (SD) or median with interquartile range (IQR), while categorical variables were presented as frequencies and percentages. The Kolmogorov–Smirnov test was used to test the normality of the distribution. The comparisons between the two groups were assessed using Student’s *t*-test for normally distributed variables and the Mann–Whitney U test for variables without normal distributions. Categorical data were compared within groups using the Chi-square or Fisher’s Exact test.

Univariate and multivariate logistic regression analyses were performed to evaluate the effects of each variable on the occurrence of left atrial thrombus. Covariates for the multivariable models were selected using a pre-specified approach based on statistical significance (*p* < 0.05 in univariate analysis) and clinical relevance. To ensure model parsimony and stability, a backward elimination process was applied. Variables demonstrating a *p*-value < 0.05 in univariate analysis were further assessed in multivariate logistic regression analysis. However, variables demonstrating multicollinearity with composite indices (e.g., components of CHA_2_DS_2_-VASc or FIB-4 formula) were excluded from the final models. Notably, left ventricular ejection fraction (LVEF) was excluded from the multivariable models to prevent multicollinearity with structural markers such as LAD and LVEDD, which were prioritized in the final models to represent the atrial and ventricular substrate. The receiver operating characteristic (ROC) curve analysis was used to determine the optimal cut-off level of admission FIB-4 values to predict left atrial thrombus. Patients were categorized into two groups according to the primary endpoint and the optimal cut-off value of FIB-4. This value was calculated with the Youden index. Spearman’s rank correlation analysis was performed to evaluate the associations of the FIB-4 index with the CHA_2_DS_2_-VASc score and the severity of spontaneous echocardiographic contrast. A *p*-value of <0.05 (using a two-sided test) was considered significant.

To further evaluate the incremental predictive value of the FIB-4 index beyond the CHA_2_DS_2_-VASc score, additional receiver operating characteristic (ROC) curve analyses were performed. The discriminatory performance of CHA_2_DS_2_-VASc alone and in combination with FIB-4 was compared using DeLong’s test. Furthermore, net reclassification improvement (NRI) analysis was conducted using a continuous (category-free) approach to assess whether the inclusion of FIB-4 improved risk stratification for left atrial thrombus.

Regarding the management of missing data, a complete-case analysis approach was adopted. Patients with missing laboratory parameters required for the calculation of the FIB-4 index or other studied variables were excluded during the initial cohort selection. Consequently, no statistical imputation was performed, ensuring that the multivariable models were based on verified clinical data.

## 3. Results

Left atrial thrombus was detected in 88 of the 859 patients (10.2%) on transesophageal echocardiography. Baseline clinical, demographic, echocardiographic and laboratory characteristics of the patients with and without thrombus are demonstrated in Table 1. A total of 488 patients (56.8%) were male, and the mean age of the study population was 57.0 ± 12.0 years old. As shown in Table 1, patients with left atrial thrombus had significantly higher LVEDD, LAD, and CHA_2_DS_2_-VASc score and higher rates of diabetes mellitus and prior stroke, as well as lower left ventricular ejection fraction, compared with those without thrombus. Regarding laboratory parameters, GFR was significantly lower, whereas AST and FIB-4 index levels were significantly higher in patients with thrombus formation (all *p* < 0.05)**.**

**Figure 1:**
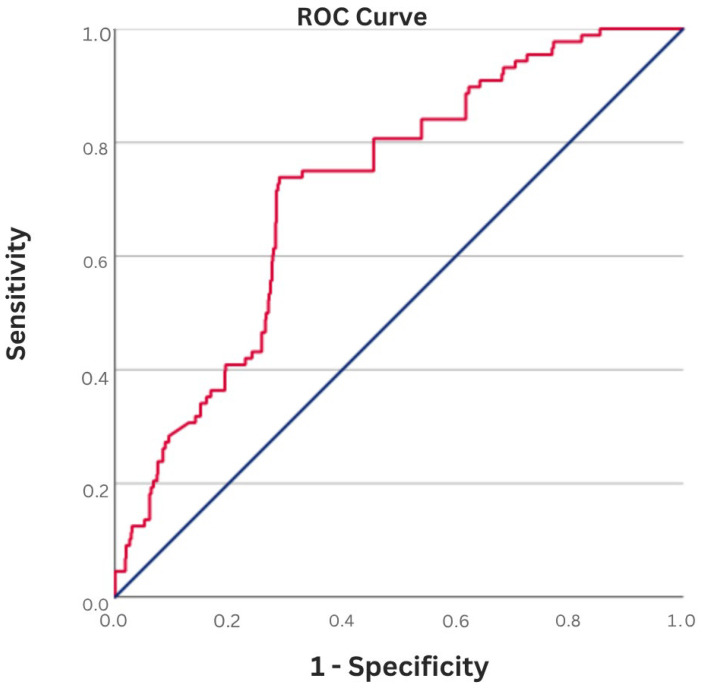
Receiver operating characteristic (ROC) curve analysis of the FIB-4 index for the prediction of left atrial thrombus in patients with nonvalvular atrial fibrillation.

Receiver operating characteristic (ROC) curve analysis demonstrated that using a cut-off value of 1.47, the admission FIB-4 index predicted left atrial thrombus with a sensitivity of 71.6% and a specificity of 72.0% (AUC: 0.65, 95% CI: 0.57–0.70, *p* < 0.001) (Figure 1). Patients were subsequently stratified into two groups according to this ROC-derived FIB-4 threshold (≥1.47).

The associations between potential risk factors and left atrial thrombus were assessed using univariate logistic regression analysis. Univariate analysis showed that diabetes mellitus, prior stroke, LVEF, LVEDD, LAD, CHA_2_DS_2_-VASc score, GFR, AST, and FIB-4 index were significantly associated with thrombus formation (for all, *p* < 0.05) (Table 2).

Subsequently, two separate multivariate logistic regression models were constructed to evaluate the predictive value of the FIB-4 index both as a continuous variable and as a dichotomized variable (FIB-4 > 1.47). After adjustment for LVEDD, LAD, CHA_2_DS_2_-VASc score, and GFR, both the continuous FIB-4 index (OR: 1.338, 95% CI: 1.018–1.757, *p* = 0.037) and FIB-4 > 1.47 (OR: 5.200, 95% CI: 3.105–8.708, *p* < 0.001) remained independent predictors of left atrial thrombus (Table 3). Although LVEF demonstrated high significance in univariate analysis, it was excluded from the final multivariable models due to the aforementioned collinearity with structural dimensions and to maintain model stability. Given the potential for multicollinearity, variables that were conceptually or statistically correlated with the main predictors (e.g., stroke and diabetes mellitus as components of CHA_2_DS_2_-VASc, and AST as a component of the FIB-4 formula) were excluded from the multivariate model despite showing significance in univariate analysis.

In addition, correlation analysis revealed a positive correlation between admission FIB-4 index and CHA_2_DS_2_-VASc score (r = 0.321, *p* < 0.001) (Table 3). Spearman’s rank correlation analysis identified a statistically significant positive relationship between FIB-4 index values and the severity of spontaneous echocardiographic contrast (r = 0.188, *p* < 0.001). Nevertheless, it is essential to note that while the associations between FIB-4 and both SEC severity and the CHA_2_DS_2_-VASc score (r = 0.321, *p* < 0.001) reached statistical significance, their overall correlation strength remains modest. This nuanced result suggests that the biological link between systemic fibrotic burden and atrial stasis, though present, may have limited direct clinical relevance in isolation and should be interpreted with caution regarding its immediate diagnostic utility.

**Figure 2:**
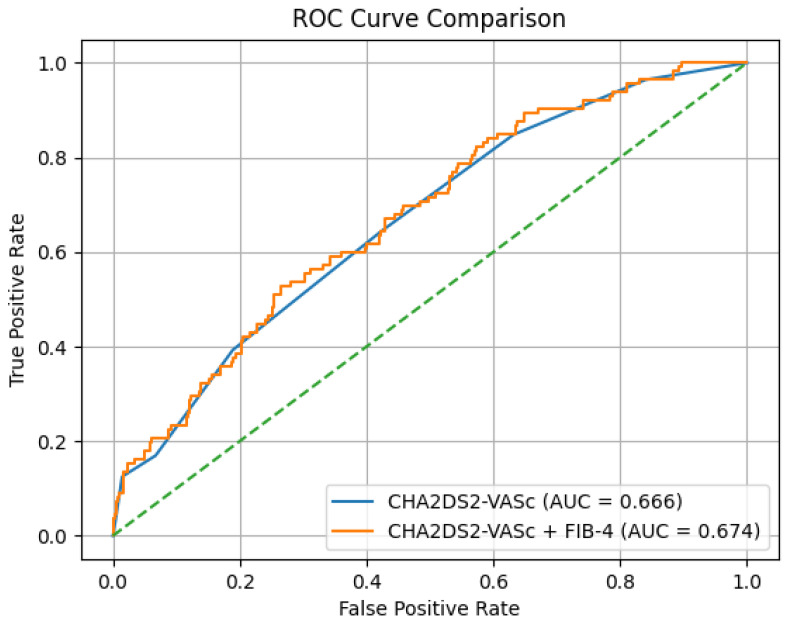
Comparison of Receiver Operating Characteristic (ROC) curves for the prediction of left atrial thrombus. The blue line represents the CHA_2_DS_2_-VASc score alone (AUC = 0.666), while the orange line represents the integrated model combining the CHA_2_DS_2_-VASc score with the FIB-4 index (AUC = 0.674).

Finally, comparing the discriminatory performance of the CHA_2_DS_2_-VASc score alone (AUC: 0.666) versus the integrated model (AUC: 0.674) showed no statistically significant difference (DeLong’s test, *p* = 0.314). Net reclassification improvement (NRI) analysis also showed no significant enhancement in risk classification with the inclusion of FIB-4 (NRI = 0) (Figure 2). Despite its modest discriminatory performance, elevated FIB-4 index values are independently associated with LAT in patients with symptomatic nonvalvular paroxysmal atrial fibrillation.

## 4. Discussion

In this study, we demonstrated that the FIB-4 index is independently associated with the presence of left atrial thrombus (LAT) in patients with nonvalvular paroxysmal atrial fibrillation undergoing transesophageal echocardiography (TEE) prior to rhythm-control interventions. Both continuous FIB-4 values and the ROC-derived cut-off of 1.47 were significantly related to thrombus formation after adjusting for established clinical risk factors, left atrial size, and renal function. Specifically, a FIB-4 index ≥ 1.47 was associated with a pronounced increase in the likelihood of LAT, suggesting the potential clinical utility of this threshold for risk stratification in early-stage arrhythmic disease.

The prevalence of LAT in our cohort was 10.2%, which is slightly higher than reported in some previous studies evaluating patients before rhythm-control interventions [3]. This difference may be explained by the overall thromboembolic risk profile of our study population, including higher CHA_2_DS_2_-VASc scores and comorbid conditions that promote atrial thrombogenesis. Importantly, all patients in this study had paroxysmal AF, suggesting that thrombus formation can occur even in the earlier stages of the disease process. Although atrial fibrillation is a well-established risk factor for ischemic stroke and systemic embolism, reliable noninvasive identification of left atrial thrombus remains challenging. Clinical risk scores such as CHA_2_DS_2_-VASc were developed to estimate long-term thromboembolic risk rather than to detect intracardiac thrombus. As a result, several studies have demonstrated limited concordance between CHA_2_DS_2_-VASc scores and the presence of left atrial thrombus or spontaneous echocardiographic contrast, underscoring the inability of clinical scores alone to fully characterize atrial thrombogenic substrate [3,16]. This limitation has driven increasing interest in adjunctive markers of atrial disease, including imaging-based and biomarker-derived indices. These studies’ analyses increasingly suggest that structural remodeling, inflammatory activation, and fibrosis play a central role in amplifying thrombotic risk, even in patients without advanced or long-standing AF [4]. Fibrosis-induced structural changes, including tissue stiffness, impaired atrial compliance, conduction heterogeneity, reduced contractility, and impaired reservoir function, have been associated with local blood stasis, spontaneous echocardiographic contrast, and LAT [6,11]. Electroanatomic mapping studies further demonstrate that low-voltage areas, representing underlying fibrosis, correlate with left atrial thrombus as well as the persistence of atrial arrhythmia [17]. Consistent with this biological framework, accumulating evidence also suggests that higher FIB-4 values correlate with atrial fibrosis, conduction abnormalities, and adverse cardiovascular outcomes, including ischemic stroke and mortality in patients with atrial fibrillation [5,9,18].

The observed association between FIB-4 and LAT is hypothesized to stem from shared pathophysiologic mechanisms involving systemic inflammation, metabolic dysfunction, and neurohumoral activation. The FIB-4 index integrates age, aminotransferase levels, and platelet-count parameters [19]. The components of the FIB-4 index collectively reflect these multisystem changes: thrombocytopenia may reflect platelet consumption or subclinical coagulopathy, while AST elevations can relate to hepatic congestion or hypoperfusion secondary to cardiovascular dysfunction. Furthermore, age serves as a shared determinant for both hepatic fibrosis and atrial remodeling. Taken together, these overlapping pathways support a theoretically plausible multisystem interaction model—a generalized fibrotic and cardio-hepatic axis—in which hepatic injury and atrial pathology reflect systemic rather than isolated organ-specific processes. However, as direct molecular or histological data were not collected, these mechanistic links remain speculative and require further prospective investigation.

In this context, the optimal FIB-4 cut-off of 1.47 identified in our study warrants careful interpretation. While this value falls within the established hepatological “intermediate risk” zone (1.30–2.67), it likely reflects an early stage of multisystem fibrotic activity and a chronic systemic fibro-inflammatory milieu rather than advanced cirrhosis [18]. In paroxysmal AF populations, this intermediate threshold may be sufficient to capture subclinical atrial remodeling and endothelial dysfunction long before clinically evident liver disease occurs. Consequently, FIB-4 may serve as a sensitive marker of systemic fibrotic burden in cardiovascular populations, even in the absence of advanced hepatic pathology.

Mechanistically, LAT formation is a multifactorial process conceptually rooted in Virchow’s triad [4]. Beyond classical hemodynamics, structural remodeling and fibrosis play a central role in amplifying thrombotic risk [6,11,17]. Our findings align with this framework, as higher FIB-4 levels showed a statistically significant but modest correlation with SEC severity (r = 0.188) and CHA_2_DS_2_-VASc scores (r = 0.321). While these correlations are weak, they suggest that FIB-4 may capture unique biological risk pathways overlooked by traditional clinical scores.

From a clinical perspective, although the discriminatory performance of FIB-4 is modest (AUC = 0.65) and it did not provide incremental predictive value beyond the CHA_2_DS_2_-VASc score in our cohort (NRI = 0, *p* = 0.314), its robust and independent predictive value in multivariable models (OR = 5.200) is noteworthy. These results suggest that FIB-4 should be viewed as a complementary, low-cost screening tool rather than a standalone diagnostic marker. Whether a biomarker-driven strategy using FIB-4 can effectively refine TEE selection and improve individualized stroke prevention remains a critical topic for future multicenter research.

## 5. Limitations

This study has several limitations that warrant consideration. Its single-center, retrospective design may introduce selection bias and limit the generalizability of the findings to a broader population. All participants were recruited from a referral-based cohort undergoing TEE prior to rhythm-control procedures, which may represent a population with higher clinical risk or symptom burden. Moreover, the absence of dedicated hepatic imaging or fibrosis-specific biomarkers precluded direct confirmation of liver fibrosis, while the lack of advanced imaging like cardiac magnetic resonance limited our insights into the myocardial fibrotic substrate.

Additionally, we lacked detailed data on important thromboembolic risk modifiers, such as quantitative AF burden and functional parameters like emptying velocity. Notably, although LAA morphology (e.g., chicken wing vs. non-chicken-wing) is a known predictor of thrombus formation, its exclusion from our multivariable models remains a limitation.

Of particular importance is the lack of granular data on anticoagulation quality. While a significant proportion of patients were receiving therapy, information regarding medication adherence, duration, and specifically the Time in Therapeutic Range (TTR) for warfarin users was missing. Since inadequate or interrupted anticoagulation is a direct driver of LAT, its absence represents a critical confounder that prevents us from definitively isolating the impact of systemic fibrosis from sub-therapeutic status. Finally, as no direct mechanistic or histological data were collected, the proposed biological pathways remain hypothesized and require prospective validation.

## 6. Conclusions

In conclusion, our study demonstrates that the FIB-4 index is independently associated with the presence of left atrial thrombus in patients with symptomatic nonvalvular paroxysmal atrial fibrillation. While the addition of FIB-4 to the CHA_2_DS_2_-VASc score did not result in a statistically significant improvement in overall discriminatory performance or net reclassification in this specific cohort, its robust and independent predictive value in multivariable models (OR: 5.200) suggests it reflects unique biological pathways related to systemic fibrotic burden. Given its ease of calculation from routine laboratory parameters, the FIB-4 index may serve as a low-cost, complementary biomarker to aid in identifying patients at higher thrombotic risk, particularly in the early stages of the arrhythmic process. However, the modest correlation with echocardiographic findings and the lack of incremental predictive power over established scores highlight its role as a supportive rather than a standalone tool. Future prospective and multicenter studies are essential to determine whether integrating FIB-4 into clinical decision-making can effectively refine TEE strategies and improve individualized stroke prevention in AF populations.

## Figures and Tables

**Table 1:** Baseline clinical, echocardiographic and laboratory characteristics of the study population stratified by the presence of left atrial thrombus.

Characteristics	Total (*n* = 859)	Left Atrial Thrombus (+) (*n* = 88)	Left Atrial Thrombus (−) (*n* = 771)	*p* Value
Age (years)	57.0 ± 12.0	58.8 ± 10.7	56.8 ± 12.1	0.159
Gender, Male *n* (%)	488 (56.8)	50 (56.8)	438 (56.8)	0.999
Hypertension, *n* (%)	534 (62.2)	61 (69.3)	473 (61.3)	0.144
Dyslipidemia, *n* (%)	343 (39.9)	43 (48.9)	300 (38.9)	0.071
Smoking, *n* (%)	175 (20.4)	22 (25.3)	153 (19.8)	0.232
Diabetes, *n* (%)	257 (29.9)	36 (40.9)	221 (28.7)	**0.017**
CAD, (%)	297 (34.6)	37 (42.0)	260 (33.7)	0.120
Stroke/TIA, (%)	48 (5.6)	10 (11.4)	38 (4.9)	**0.013**
COPD, (%)	49 (5.7)	7 (8.0)	42 (5.4)	0.337
CKD, (%)	70 (8.1)	11 (12.5)	59 (7.7)	0.115
Aspirin, *n* (%)	115 (13.4)	17 (19.3)	98 (12.7)	0.085
P2Y12 inh, *n* (%)	39 (4.5)	4 (4.5)	35 (4.5)	0.998
Warfarin, (%)	123 (14.3)	18 (20.5)	105 (13.6)	0.083
Apixaban, *n* (%)	184 (21.4)	17 (19.3)	167 (21.7)	0.612
Rivaroxaban, *n* (%)	207 (24.1)	22 (25.0)	185 (24.0)	0.835
Edoxaban, *n* (%)	87 (10.1)	10 (11.4)	77 (10.0)	0.685
Dabigatran, *n* (%)	9 (1.0)	0 (0.0)	9 (1.2)	0.308
Left atrial diameter (mm)	43.4 ± 4.3	46.0 ± 5.3	43.1 ± 4.1	**<0.001**
LVEF, %	52.6 ± 11.6	43.9 ± 15.0	53.6 ± 10.8	**<0.001**
LVEDD, mm	48.6 ± 5.5	51.0 ± 8.3	48.4 ± 5.0	**<0.001**
SPAP, mmHg	30.3 ± 7.0	31.3 ± 4.6	30.2 ± 7.2	0.170
CHA_2_DS_2_-VASc score	2.3 ± 1.5	3.2 ± 1.5	2.2 ± 1.5	**<0.001**
Glucose (mg/dL) ^a^	99 (89–121)	103 (89–140)	99 (89–119)	0.059
Uric acid, mg/dL	5.8 ± 1.6	5.7 ± 1.6	6.1 ± 1.6	0.074
Urea ^a^ (mg/dL)	35 (30–42)	36 (30–43)	35 (30–41)	0.288
Creatinine ^a^ (mg/dL)	0.9 (0.8–1.0)	0.9 (0.8–1.0)	0.9 (0.7–1.0)	0.091
GFR, mL/min/1.73 m^2^	83 ± 17	77 ± 19	84 ± 17	**<0.001**
AST, U/L	28 ± 17	32 ± 17	27 ± 17	**0.018**
ALT, U/L	31 ± 28	33 ± 24	30 ± 28	0.417
NT-pro BNP (ng/L)	223 ± 105	238 ± 123	221 ± 102	0.143
Triglyceride (mg/dL)	162 ± 81	147 ± 68	164 ± 83	0.065
LDL-C (mg/dL)	104 ± 33	99 ± 44	105 ± 31	0.147
HDL-C (mg/dL)	42 ± 11	41 ± 12	42 ± 11	0.288
Albumin (mg/dL)	4.2 ± 0.4	4.2 ± 0.4	4.2 ± 0.4	0.179
hsCRP (mg/dL)	10.9 ± 11.7	12.7 ± 11.5	10.6 ± 11.8	0.121
Hemoglobin (g/dL)	13.5 ± 1.8	13.4 ± 1.9	13.5 ± 1.8	0.506
WBC (×10^3^ µL)	7.6 ± 2.0	7.9 ± 2.1	7.6 ± 2.0	0.174
Neutrophil (×10^3^ µL)	4.6 ± 1.6	4.5 ± 1.6	4.7 ± 1.6	0.340
Lymphocyte ^a^ (×10^3^ µL)	2.1 ± 0.7	1.9 ± 0.8	2.1 ± 0.7	0.087
Platelet, (×10^3^ µL)	245 ± 65	233 ± 84	247 ± 62	0.620
FIB-4 index ^a^	1.1 (0.8–1.6)	1.5 (0.9–2.0)	1.1 (0.8–1.6)	**<0.001**

Data are presented as mean ± SD or *n* (%). CAD, coronary artery disease; CHA_2_DS_2_-VASc, congestive heart failure, hypertension, age ≥ 75 (2 points), diabetes mellitus, stroke/TIA (2 points), vascular disease, age 65–74, sex category CKD, chronic kidney disease; COPD, chronic obstructive pulmonary disease; GFR, glomerular filtration rate; HDL-C, high-density lipoprotein cholesterol; hsCRP, high-sensitivity C-reactive protein; LVEF, left ventricular ejection fraction; SD, standard deviation; TIA, transient ischemic attack; WBC, white blood cell count. Bold data displays a statistically significant difference (*p* < 0.05). ^a^ Comparison was made using the Mann–Whitney U test at *p* < 0.05, and these values were described by median with inter-quartile range (25th and 75th percentile).

**Table 2:** Univariate logistic regression analysis for prediction of left atrial thrombus formation.

Variable	OR	95% CI	*p* Value
Age	1.014	0.995–1.034	0.160
Gender	1.000	0.640–1.560	0.999
Hypertension	1.423	0.885–2.290	0.146
Dyslipidemia	1.500	0.964–2.335	0.072
Smoking	1.367	0.817–2.288	0.234
Diabetes mellitus	1.723	1.096–2.709	**0.019**
CAD	1.494	0.955–2.337	0.079
Stroke/TIA	2.473	1.186–5.156	**0.016**
COPD	1.500	0.652–3.448	0.340
CKD	1.724	0.869–3.421	0.119
Aspirin	1.644	0.930–2.908	0.087
P2Y12 inh	1.001	0.347–2.887	0.998
Warfarin	1.631	0.934–2.848	0.085
Apixaban	0.866	0.497–1.510	0.612
Rivaroxaban	1.056	0.634–1.758	0.835
Edoxaban	1.156	0.574–2.325	0.685
Left atrial diameter	1.144	1.091–1.200	**<0.001**
LVEF (%)	0.947	0.933–0.962	**<0.001**
LVEDD (mm)	1.074	1.038–1.111	**<0.001**
SPAP (mmHg)	1.021	0.991–1.051	0.170
CHA_2_DS_2_-VASc score	1.522	1.317–1.758	**<0.001**
Glucose	1.003	0.998–1.008	0.224
Uric acid	1.123	0.989–1.276	0.075
Urea	1.008	0.990–1.026	0.391
Creatinine	2.144	0.858–5.355	0.102
GFR	0.979	0.967–0.991	**<0.001**
AST	1.011	1.001–1.021	**0.039**
ALT	1.003	0.996–1.009	0.423
Nt-pro BNP	1.002	0.999–1.004	0.144
Triglyceride	0.997	0.994–1.000	0.067
LDL-C	0.995	0.988–1.002	0.147
HDL-C	0.989	0.970–1.009	0.288
Albumin	0.969	0.925–1.015	0.179
hsCRP	1.013	0.996–1.030	0.123
Hemoglobin	0.960	0.853–1.082	0.505
WBC	1.073	0.969–1.188	0.175
Neutrophil	0.933	0.809–1.076	0.340
Lymphocyte	0.754	0.547–1.039	0.084
FIB-4 index	1.555	1.220–1.982	**0.006**

Bolded values indicate statistical significance (*p* < 0.05). CAD, coronary artery disease; CHA_2_DS_2_-VASc, congestive heart failure, hypertension, age ≥ 75 (2 points), diabetes mellitus, stroke/TIA (2 points), vascular disease, age 65–74, sex category CKD, chronic kidney disease; COPD, chronic obstructive pulmonary disease; CI, confidence interval; GFR, glomerular filtration rate; HDL-C, high-density lipoprotein cholesterol; hsCRP, high-sensitivity C-reactive protein; LVEDD, left ventricular end-diastolic diameter; LVEF, left ventricular ejection fraction; OR, odds ratio; TIA, transient ischemic attack; WBC, white blood cell count.

**Table 3:** Multivariate logistic regression analyses to examine the association between FIB-4 index and left atrial thrombus.

Variables	Adjusted OR	95% CI	*p*-Value
**MODEL 1**			
CHA_2_DS_2_-VASc score	1.387	1.182–1.629	**<0.001**
LAD	1.113	1.055–1.174	**<0.001**
LVEDD	1.029	0.990–1.069	0.150
GFR	0.992	0.979–1.006	0.273
FIB-4	1.338	1.018–1.757	**0.037**
**MODEL 2**			
CHA_2_DS_2_-VASc score	1.327	1.120–1.572	**0.001**
LAD	1.121	1.061–1.184	**<0.001**
LVEDD	1.029	0.988–1.070	0.167
GFR	0.996	0.981–1.010	0.565
FIB-4 > 1.47	5.200	3.105–8.708	**<0.001**

CI, confidence interval; FIB-4, fibrosis-4 index; GFR, glomerular filtration rate; LAD, left atrial diameter; LVEDD, left ventricular end-diastolic diameter; OR, odds ratio; CHA_2_DS_2_-VASc, congestive heart failure, hypertension, age ≥ 75 (2 points), diabetes mellitus, stroke/TIA (2 points), vascular disease, age 65–74, sex category. Bolded values indicate statistical significance (*p* < 0.05).

## Data Availability

The data presented in this study are available on reasonable request from the corresponding author. The data are not publicly available due to privacy and ethical restrictions.
